# Reduced Graphene Oxide Aerogels Cartridges for Solid Phase Extraction of Benzotriazoles

**DOI:** 10.3390/ma16062519

**Published:** 2023-03-22

**Authors:** Samantha L. Flores-López, Ana Arenillas, Ivan Mikšík, J. Angel Menéndez, Miguel A. Montes-Morán

**Affiliations:** 1 Instituto de Ciencia y Tecnología del Carbono, INCAR-CSIC, Francisco Pintado Fe, 26, 33011 Oviedo, Spain; 2 Institute of Physiology, The Czech Academy of Sciences, 142 20 Prague, Czech Republic

**Keywords:** reduced graphene oxide aerogels, solid phase extraction, SPE, UV-benzotriazoles

## Abstract

UV-benzotriazoles have been identified as water micropollutants that cause serious problems for human health and the environment. Their low concentration in water bodies complicates their detection by direct water analysis, slowing the corrective actions to avoid bioaccumulation. In this regard, the use of graphene-based materials with a high affinity for non-polar molecules has been demonstrated to be a potential tool for the optimal separation and concentration of this type of molecules in solid phase extraction (SPE) processes. This work evaluates the potential of novel reduced graphene oxide aerogels (rGO) as extractants of mixtures of three UV-benzotriazoles in water at low concentrations. These rGO aerogels incorporate graphenic domains into a tough structure of polymeric chains by adding graphene oxide during the synthesis of resorcinol-formaldehyde gels. Aerogels with a different content and ordering of graphenic domains were obtained and characterized using Raman, XRD, SEM and nitrogen adsorption isotherms (−196 °C). The rGO aerogels that performed better as solid phase extractants were those containing 60% rGO. Aerogels with lower rGO contents (40%) required a high-temperature (2000 °C) treatment to render competitive results. The SPE methodology using selected rGO aerogels was optimized by varying the elution solvent, elution time and volume. The best performances, i.e., recoveries of 80–100% and enrichment factors of 12.5–50, were accomplished when using 0.8 mL of tetrahydrofuran (THF) as an elution solvent. As a result, a fast (10 min) and simple extraction method of UV-benzotriazoles in water was attained, achieving a detection limit of 1 ng mL^−1^. Selected aerogels were finally tested for the SPE of spiked samples of river waters, showing a similar performance to that observed with synthetic mixtures.

## 1. Introduction

Water pollution is an environmental problem that demands urgent actions for remediation. The presence of heavy metals, industry chemicals, domestic wastes, pathogens, oxidizable materials, etc. in water bodies causes a considerable threat to human health and water availability [1,2]. The harmful effect and origin of all these species have been deeply studied, and processes applied in wastewater treatment plants have been designed for their successful removal. However, in recent decades, major attention has been focused on micropollutants, which are found in aquatic environments in small concentrations, from nanograms to micrograms per liter [3,4]. Their presence, even in such small amounts, affects water ecosystems by chronic exposure, bioaccumulation and long-term toxicity [2,5]. Among micropollutants, molecules derived from pharmaceuticals and personal care products are considered relevant species due to their adverse effects on living organisms [5,6]. Such is the case of UV-benzotriazoles, a family of emerging micropollutants used for UV protection in cosmetics. They are also present in a wide variety of products such as adhesives, plastics, coatings, automotive parts, packaging, paints, textiles, glazing materials, etc., which contributes to their spreading. UV-benzotriazoles are readily adsorbed molecules that accumulate in tissues, damaging collagen and elastin, acting as endocrine disruptors and promoting mutagenic effects in humans and other living organisms [7,8]. These compounds are resistant to biological and chemical degradation, thus withstanding the common wastewater treatment processes, which complicates their removal once they are disposed of in the environment [8].

For the risk assessment of these compounds in aquatic ecosystems, several methods have been proposed for extracting UV-benzotriazoles from flora and fauna tissues. However, challenges related to the correct analysis of these molecules directly at the source of the problem (i.e., water) limit the information available on their critical concentrations or possible transformation into more hazardous compounds [3,6,8]. This strongly complicates the definition of parameters for the regulation on UV-benzotriazoles and the development of treatments for their removal [9]. The situation makes the improvement of analytical methods for the rapid and selective quantification of UV-benzotriazoles as specific target molecules urgent. In this regard, extraction processes stand out in separating and concentrating molecules of interest from real and complex samples. In comparison with liquid–liquid extraction (LLE), solid phase extraction (SPE) is preferred due to its high selectivity, low cost, time saving and easy adaptability to analytical techniques [4,10,11,12]. As the comparative works of Leusch et al. [13] and Guillot et al. [14] show, the SPE of micropollutants from water results in lower variability, higher extraction efficiency (twice for some analytes) and a higher concentration (up to 200 times) than LLE, using 97% less solvent volume and a competitive extraction time (ca. 30 min) and avoiding the formation of emulsions.

SPE is useful for the isolation of compounds from environmental water samples with highly complex organic matrixes and a low concentration of the analytes of interest due to the selective interaction with the solid stationary phase. In a typical SPE protocol, the solid stationary phase is first conditioned. Then, a solution of interest is put in contact with this solid capable of retaining the target molecules for loading. The loaded solid is washed to remove unwanted analytes and dried. In the final step, the elution of the target molecules is performed with an organic solvent. SPE is always coupled with an analytical technique, most often chromatography, for the separation and quantification of the target analytes from the eluent. The performance of SPE is evaluated by means of two factors: the recovery percentage and the enrichment factor (EF). The recovery percentage indicates the quantity of target molecules extracted from the original solution, i.e., 100% recovery percentage would mean that all the molecules present in the original solution have been successfully extracted to the organic eluent. It is related to the retention and elution capacities of the solid used and it is affected by the matrix that contains the analytes. The retention and elution capacities can be determined by using solutions of known concentrations in pure solvents (i.e., standard solutions), whilst effects of the matrix are determined by spiking known concentrations of target compounds and comparing with the results of the standard solutions [15,16]. Typical recoveries of 60–100% are reported in the SPE methods [10,17]. On the other hand, the EF represents the level of concentration reached by the method, and it is calculated as the ratio of the sample volume divided by the volume of the elution solvent used. EF is especially relevant, as SPE is not only intended to isolate the target molecules from a given matrix but also to concentrate the analytes, which is very significant when working with micropollutants in water. In that latter issue, the entire methodology is also affected by the sensitivity of the analytical procedure, which defines the limit of detection (LOD) [12]. Finally, SPE can also be used to improve this LOD by means of a transfer of the analytes to a solvent more compatible with the detection technique. Different methods have been developed from conventional SPE, including micro-SPE, dispersive-SPE or magnetic-SPE, to reduce the consumption of solvents and to increase the concentration of the analytes. However, the conventional, cartridge-based SPE procedures still stand out because they are economic and due to their simple handling, which facilitates their use in field sampling and subsequent automatization, which is the most popular approach in high-throughput industrial SPE [11,12].

Regarding the materials involved in SPE (aka extractants), it should be clear that, in order to perform good extractions, the most important feature lies in the interaction of the solid material with the molecules of interest, as this defines the selectivity of the method [10]. Additionally, good stability and permeability are also requirements for good extractants. Stability will allow for the use of a wide range of conditions (i.e., solvent, pH and temperature) to improve the extraction performance while keeping good reproducibility, thus avoiding the effects of matrix interferences. A good permeability guarantees maximum access to the extractant surface, thus providing more sites for extraction.

Porous materials based on graphene/reduced graphene oxide (rGO) have shown good extraction performances for emerging organic pollutants, including UV-benzotriazoles. This is due to their high surface area combined with graphene-like nanostructures that promote interactions with the organic molecules based on π stacking, H-bonds and dipole forces [4,11]. In addition, other properties such as thermal stability, good adsorption–desorption kinetics, easy regeneration, functionalization and good compatibility with many solvents make graphene-based materials a very attractive option as extractants [18]. Therefore, graphene sponges (GSs) have been applied for the extraction of different micropollutants such as pesticides [19], endocrine disruptors [20] and UV-benzotriazoles [21]. In the work of Wang et al. [21], a GS was synthesized by the direct lyophilization and subsequent reduction of a GO suspension. With this GS, the authors reached recoveries of about 90% for six different benzotriazoles extracted from cosmetics, showing a clear advantage of GS over other separation materials such as carbon nanotubes or C18 columns. However, the small size and light weight of the rGO sheets limit the real application of these GSs, especially under high-pressure conditions [22]. To overcome these drawbacks, composite materials containing graphene/rGO have been recently explored [22,23,24], including a nickel foam coated with graphene for the extraction of UV-benzotriazoles [22].

In this work, a new alternative to GSs for the extraction of UV-benzotriazoles in water solutions is explored. rGO aerogels (GAs) obtained through the sol-gel process by the addition of GO suspensions in gelling resorcinol-formaldehyde resins are suggested as a novel graphene-based composite material that averts the shortcomings of GSs. The synthesis of the GAs is a relatively simple and time-saving process. Furthermore, the composition of the aerogels can be modulated, with a controlled impact on their porous properties, which in turn influence their performance as solid extractants. Compared to GSs, the possibility of these modifications allows for the optimal design of GAs for their application in SPE.

## 2. Materials and Methods

### 2.1. Materials

Resorcinol (R, Indspec, Pittsburgh, PA, USA, 99%), formaldehyde (F, Merck, New York, NY, USA, 37%) and graphene oxide (GO) suspension (Applynano Solutions S.L, Alicante, Spain) were used for the synthesis of the GAs and GS. For the SPE tests, the following UV-benzotriazoles were selected as representative of this molecular family: 2-(2′-hydroxy-5′-methylphenyl)benzotriazole (UVP, Sigma Aldrich, St. Louis, MO, USA, 97%), 2-(2-hydroxy-5-tert-octylphenyl)benzotriazole (UV329, Sigma Aldrich, 98%) and 2-(2*H*-benzotriazol-2-yl)-4,6-bis(1-methyl-1-phenylethyl)phenol (UV234, Sigma Aldrich, 100%). Acetonitrile (ACN, LiChrosolv Merck, Darmstadt, Germany, 99.9%), acetone (ACE, Fisher scientific, Freiburg, Germany, 99%), tetrahydrofuran (THF, Aldrich chemicals, Merck, Darmstadt, Germany, 99.9%) and Milli-Q^®^ (Merck, Darmstadt, Germany) water were used as solvents in the elution tests and HPLC analyses.

### 2.2. Synthesis and Characterization of the rGO Aerogels

**Figure 1:**
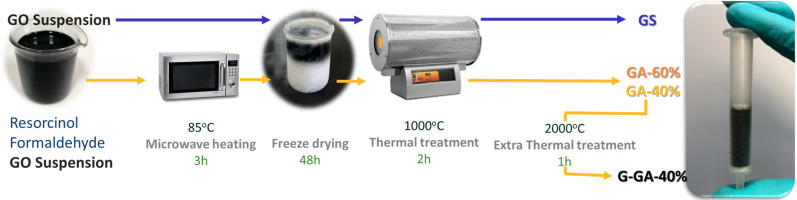
Synthesis of the rGO aerogels (GAs) via sol-gel processing and of the graphene sponge (GS). All times correspond to the dwell times at the set temperature.

rGO aerogels were synthesized by the sol-gel process, incorporating rGO sheets into a resorcinol-formaldehyde (RF) polymeric network [19]. Precursor solutions were prepared from R and F mixtures, using the GO suspension as the solvent. The quantities of each compound added were calculated considering pure water as the solvent, with the R/F molar ratio fixed at 0.5 and the dilution ratio (D), defined as the molar ratio between the total solvent and the reactants, fixed at 200. The final rGO is thus incorporated in the aerogels by replacing the calculated volume of water with the GO suspension (in water). Two different concentrations of the GO suspension (i.e., 5 and 10 mg L^−1^) were employed to adjust the rGO content on the aerogels. Polymerization was carried out under microwave heating at 85 °C for 3 h. Subsequently, freeze drying (48 h) was performed to avoid the collapse of the structure. Additionally, a GO sponge was prepared by direct lyophilization of the graphene oxide suspension (5 mg L^−1^) and used for comparison. The dried gels and the sponge were treated under N_2_ atmosphere at 1000 °C for 2 h to carbonize the RF network and/or to reduce the GO. The final rGO content in the aerogels was calculated from the mass balance of the entire synthesis process, assuming that all of the GO is totally reduced [19], obtaining 40 and 60 wt.% rGO contents when the 5 and 10 mg L^−1^ GO suspensions were used in the synthesis of the aerogels, respectively. The reduced sponge was labeled as GS, and the aerogels with different rGO contents were labeled as GA-40% and GA-60%. A third sample was prepared by the additional heat treatment of the sample with the lower rGO content (i.e., GA-40%) at 2000 °C for 1 h in an inert atmosphere, denoted as G-GA-40%. The entire synthesis process is presented in Figure 1.

Structural analysis of the materials was performed by means of X-ray diffraction (XRD) analysis and Raman spectroscopy. A D8 Advance Bruker diffractometer (Karlsruhe, Germany) with CuKα radiation operated at 40 kV and 40 mA was used, whilst for the Raman spectra acquisition, a Renishaw inVia Qontor spectrometer (Gloucestershire, UK) equipped with an Nd:YAG (diode pumped solid state) laser (532 nm) and a deep depletion CCD detector was used. The morphology of the samples was characterized with a Quanta FEG 650 SEM (FEI, Hillsboro, OR, USA) working with an accelerating voltage of 20 kV and a secondary electron detector (EDT, Everhart–Thornley). Finally, porous characterization was performed by N_2_ adsorption–desorption isotherms (−196 °C) recorded in a Tristar II from Micromeritics (Norcross, GA, USA). Before the textural analysis, the samples were dried at 120 °C under vacuum for 8 h. Specific surface areas (S_BET_) and micropore volumes (V_micro_) were determined using de Brunauer–Emmett–Teller and Dubinin–Radushkevich equations, respectively.

### 2.3. Solid Phase Extraction Tests

Mixtures of the three UV-benzotriazoles at different concentrations (i.e., 1, 5, 50 and 100 ng mL^−1^) were prepared using Milli-Q^®^ water as a solvent to evaluate the extraction capacity of the materials. For SPE experiments, 15 mg of a given material was placed into polypropylene cartridges and compressed between two frits down to an approx. 0.7 mm bed height to avoid channeling effects. Prior to use, the cartridges were washed with 20 mL of water and 10 mL of solvent. For the recovery and optimization tests, 10 mL of a 100 ng mL^−1^ UV-benzotriazole solution was first loaded into the cartridge. Then, the loaded UV-benzotriazole was eluted using 0.8 mL of the elution solvent. ACN, ACE and THF were tested as elution solvents. The recovery percentage was optimized by means of the extraction time (i.e., 1 or 10 min) and the volume of the solvent used (i.e., 0.4, 0.6 or 0.8 mL). In addition, the possibility of evaporating the elution solvent and redissolving the recovered UV-benzotriazoles was studied as an alternative to increase the enrichment factor achieved by the method, using 32 and 50 °C as evaporation temperatures. Materials with the best performance were used for the extraction of the UV-benzotriazoles from aqueous solutions with initial concentrations of 1–100 ng mL^−1^. All SPE tests were carried out at least in triplicate.

The reuse of the selected GA-60% and G-GA-40% cartridges was carried out by extracting aqueous solutions of benzotriazoles mixtures (100 ng mL^−1^) three consecutive times. The cartridges were cleaned after each use by eluting with 5 mL of THF, drying (room temperature), eluting with 10 mL of Milli-Q^®^ water and drying (105 °C).

The carryover effect was estimated by eluting 0.8 mL of THF through G-GA-40% cartridges previously used for the SPE of a mixture of UV-benzotriazoles in water (100 ng mL^−1^). Additional solvents were used to evaluate the carryover effect of UVP.

Finally, the evaluation of real samples was carried out with a water sample collected from the Nora river close to Lugones (Asturias, Spain). The water sample was filtered (0.45 µm PTFE) and used for the preparation of samples spiked with mixtures of the three benzotriazoles (25, 50 or 100 ng mL^−1^) in river water.

### 2.4. Chromatographic Conditions

The UV-benzotriazoles were analyzed with an HPLC-UV Agilent 1100 Series, using the Kinetex 2.6 µm PFP, 100 Å, 150 mm × 2.1 mm column (Phenomenex, Torrance, CA, USA). The column temperature was set at 25 °C. A mixture of H_2_O:ACN was used as the mobile phase using gradient elution. The gradient started with a composition of 80:20 (H_2_O:ACN, *v*/*v*) and a flow rate of 0.25 mL min^−1^; then, the ACN concentration increased linearly to 100% in 30 min and returned to the initial 80:20 H_2_O:ACN composition in two minutes. A total running time of 60 min was employed to allow for the re-equilibration of the column. Analytical features of the HPLC method (standards in THF) are detailed in Table S1.

## 3. Results and Discussion

### 3.1. Properties of the rGO Aerogels

The rGO aerogels were studied in the present work as a possible upgrade of the GSs for SPE [19,20,21] on the basis of the GAs properties found in our recent work [25]. Specifically, GAs had much better mechanical properties than GS, which was quite difficult to handle. Considering this, we started with the synthesis of the GAs with the highest rGO content (60%) that preserves that mechanical integrity. We then reduced the content to 40% and found that the performance of the resulting aerogel as an SPE extractant for this particular application was quite poor (see below). In searching for an improved surface with extended graphenic/graphitic domains, we decided to test the effects of increasing the crystallinity of the GA-40% by a high-temperature treatment at 2000 °C (Figure 1).

**Figure 2:**
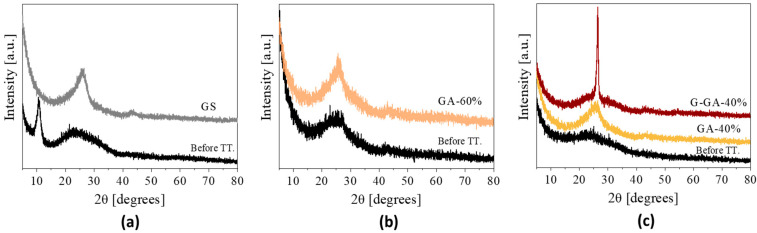
XRD diffraction of the graphene-based materials before and after the thermal treatment (TT.) at 1000 °C, 2 h in inert atmosphere: (**a**) GS; (**b**) GA-60%; (**c**) GA-40%, also including the pattern of G-GA-40%.

Through the structural characterization, the reduction in GO and the presence of ordered domains in the structure of the materials were evaluated. To confirm the conversion of GO to graphene under thermal reduction, XRD analysis was performed before and after the thermal treatments (Figure 2). The diffraction patterns of the materials before thermal treatment present differences based on their composition. In the case of the sponge (Figure 2a), a well-defined peak at 10.8° (2θ) and a broad one at about 23.7° (2θ) indicate the presence of oxygen functionalities in the interlayer space of GO and zones with a slightly high ordering and a lower content of oxygenated groups, respectively [26]. For the aerogels (Figure 2b,c), the characteristic GO peak at 10.8° (2θ) is missing in the freeze-dried (i.e., before thermal treatment) materials, probably because of a different distribution of the GO sheets and the partial reduction in oxygen functional groups promoted by the presence of the RF polymeric structures. Therefore, only the broad peak at 23.7° (2θ) can be appreciated in those patterns. On the other hand, all the thermally treated samples showed a better-defined band at 25° (2θ) (Figure 2a–c), which corroborates the reduction in GO [25,26,27]. The GO sheets present during the gelling of the RF network are prone to crosslink via covalent bonding through the GO oxygen functionalities. This has been illustrated in our recent publication [25]. During the carbonization, these covalent bonds will either break with the emission of volatiles or be transformed into sp^2^ or sp^3^ carbon structures. As a result, a reduction in the GO is expected, with an almost complete removal of heteroatoms (mainly O) and the partial unsaturation of the carbon bonds in the rGO layers. Finally, the high-temperature (2000 °C) thermal treatment of the sample G-GA-40% contributes to the formation of structures with better crystallinity, showing a well-defined peak at 26.5° (2θ) (Figure 2c).

**Figure 3:**
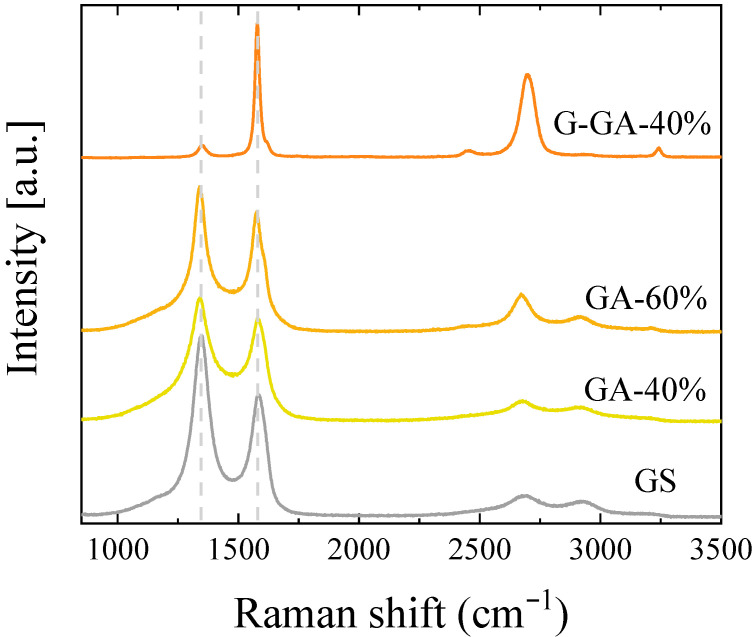
Raman spectra of the graphene-based materials.

Raman analysis of the GS and GAs also confirmed the presence of relatively ordered carbon structures (Figure 3). The Raman spectra of the GA-40%, GA-60% and GS are relatively similar, with the two main D and G first-order bands overlapping. In the case of GA-60%, the G band at 1580 cm^−1^ has an incipient shoulder, which would suggest better ordered domains in this particular sample. Also for this sample, the second-order bands are better defined when compared to those of GS and GA-40%. As expected from the XRD results, the high-temperature treatment of GA-40% (sample G-GA-40%) strongly reduces the intensity of the D band at 1330 cm^−1^ and increases the intensity of the second-order bands, thus suggesting the presence of highly ordered carbon structures. So, regardless of the amount of GO used in this work for the synthesis of the graphene-containing materials, the presence of well-ordered sp^2^ carbon domains in all of them is confirmed.

**Figure 4:**
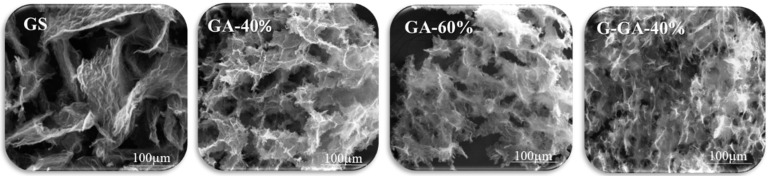
Representative SEM micrographs of the graphene-based materials.

While the use of GO in the synthesis of the aerogels contributes to the development of graphenic domains, the incorporation of RF species mainly impacts the morphology through the formation of a polymeric network. As shown in Figure 4, the sponge-like morphology of GS considerably differs from that of the GAs. The GS presents rGO sheets of a relatively big size in a random distribution that creates large empty spaces between them. Although the porosity formed in this structure is useful, its arrangement makes it extremely fragile, thus limiting its application [18,28]. On the other hand, the GAs have a better-defined porous structure attributed to the presence of RF species, which react with functionalities of the GO sheets acting as a crosslinking agent, providing resistance to the material obtained and benefiting their handling [25]. Among these aerogels, no morphological differences were found with the variation in the rGO content between 40% and 60% (see GA-40% and GA-60% in Figure 4). Furthermore, even after the high-temperature thermal treatment, the morphology of G-GA-40% remains almost unaltered.

**Figure 5:**
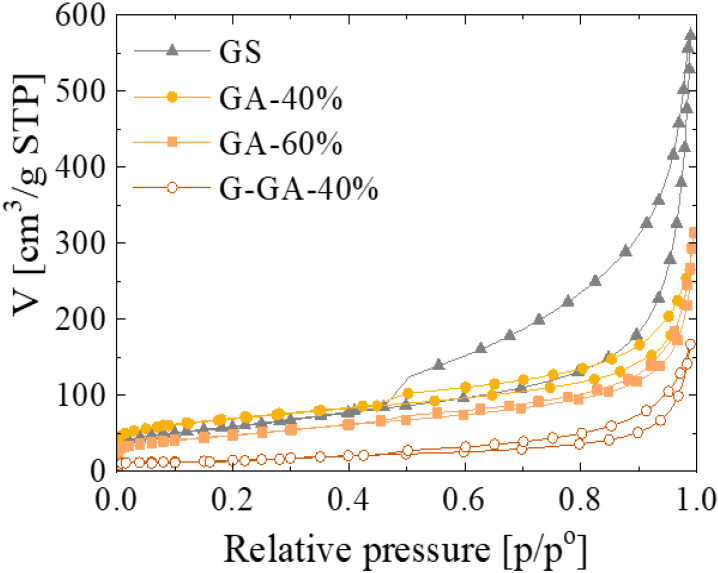
N_2_ adsorption–desorption isotherms of the graphene-based materials.

Regardless of morphological differences, the aerogels synthesized in this work preserve an open pore structure that can be competitive with that of sponge-like materials. For further analysis, textural properties were also evaluated by nitrogen adsorption at −196 °C. Figure 5 shows the N_2_ isotherms of the GAs and the GS. All the materials present a type II isotherm characteristic of macroporous materials. However, different N_2_ adsorption behavior is appreciated at both high and low relative pressures depending on the composition of the graphene-based material, either aerogel or sponge. In the higher relative pressure range, the presence of a hysteresis cycle is attributed to the incomplete filling of the mesopores, and the occurrence of a particular hysteresis cycle shape depends on the different pore characteristics influencing the effect of N_2_ condensation [29]. Thus, whilst the differences between the adsorption and desorption branches in GAs is small, the big hysteresis cycle in the GS isotherm could be attributed to a particularly strong N_2_ condensation effect on this sample due to a multilayer adsorption on the rGO sheets. As shown in Figure 4, the rGO sheets present in GS would be (i) more accessible and (ii) of a bigger size than those of the GAs for multilayer N_2_ adsorption. A direct consequence of that effect is the relatively high amount of N_2_ adsorbed on this sample, expressed as the total pore volume or V_total_ (Table 1). On the other hand, the N_2_ adsorbed on the GAs in the lower relative pressure range changes with their rGO content and the thermal treatment applied. These variations are better quantified by the specific surface area (S_BET_) and micropore volume (V_micro_) values shown in Table 1. During the carbonization process of the RF polymeric nodules present in the GAs, the loss of oxygen functionalities generates micropores [30,31]. Hence, when the rGO content is lower, a higher quantity of polymeric clusters contributes to a higher specific surface area of the corresponding material. In other words, the lower the rGO content in the GA, the higher its V_micro_ and S_BET_ (see GA-40% and GA-60% in Table 1). Furthermore, as was demonstrated by XRD, the use of higher temperatures to prepare G-GA-40% favored the ordering of the microstructure, which in textural terms means a significant reduction in the micropore volume and specific surface area of the material [27,32]. On its part, the S_BET_ and V_micro_ values for GS do not follow the expected trend based on its rGO content. The values obtained for this sample are, in fact, overestimated due to the strong N_2_ multilayer adsorption on this particular material, as discussed above.

In sum, all GAs and GS have adequate properties for the desired application, that is, presence of graphenic domains and open meso/macropores. Among aerogels, GA-60% is the material showing intermediate properties, which, in addition, can be considered similar to the reference material GS.

### 3.2. rGO Aerogels in Solid Phase Extraction

#### 3.2.1. Recovery Test: Elution Solvent and rGO Aerogel Performance

**Figure 6:**
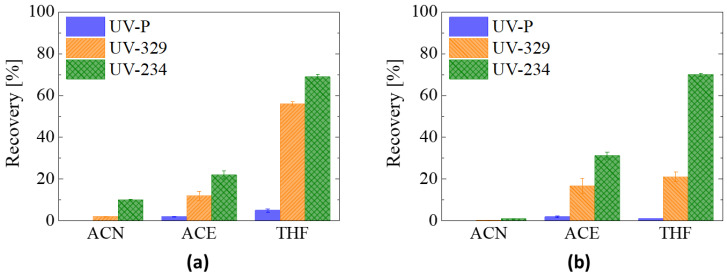
Extraction performance of different elution solvents (n = 3) for (**a**) GS and (**b**) GA-60%.

GS, as the reference material, and GA-60%, as the representative rGO aerogel, were selected to perform the evaluation of different elution solvents. These tests were carried out using polar aprotic solvents since they have shown good affinity for UV-benzotriazoles in previous works [21,23,33,34,35]. Specifically, three solvents with different dipole moment—acetonitrile (ACN, 3.92 D), acetone (ACE, 2.8 D) and tetrahydrofuran (THF, 1.75 D)—were chosen. A fast extraction process (approx. 1 min) was performed by eluting the benzotriazoles using the cartridge as a syringe filter. Figure 6 shows the recoveries calculated for each solvent. All three UV-benzotriazoles show an increase in the recovery percentage as the dipole moment of the solvent decreases. For example, the recoveries of UV329 in the GS are 2%, 12% and 48% for ACN, ACE and THF, respectively. Although the recovery tests performed with GA-60% show a similar trend, the values achieved for the smaller benzotriazoles are lower than those of GS, a behavior that is related to the contribution of the carbon clusters (from the RF carbonization) in the aerogel (see below).

It should be noted that, for the same material and regardless of the solvent used, the recovery values differ considerably from one benzotriazole to another, 10% and 69% being the maximum recoveries achieved when using GS with THF for UVP and UV234, respectively. Such variation can be understood based on the polarity of the UV-benzotriazole molecules and their interaction with the graphenic structures in the adsorbent material. Using the octanol-water partition coefficient (LogK_ow_) as a measure of the polarity of organic molecules, the majority of UV-benzotriazoles have values between 4.3 and 8.0 [33,36], with two of the molecules selected in this work nearest to the bottom and top limits of the mentioned LogK_ow_ range (i.e., 4.3 and 7.7 for UVP and UV234, respectively). On the other hand, the retention of aromatic molecules on graphenic materials is mainly based on π-π and hydrophobic interactions [18,19,37]; hence, the interaction with non-polar compounds is highly favored. The molecule with the lowest polarity (i.e., UV234) shows the highest recovery values, whilst for the most polar one (i.e., UVP), considerably lower recoveries were achieved (Figure 6). Moreover, UVP molecules seem to have difficulties interacting properly with the materials under the test conditions used. The microporosity of the materials could also be influencing the extraction yield of UV-benzotriazoles by acting as irreversible sorption sites, limiting the elution capacity of mainly small molecules—in this case, again, UVP (Table S2). Nevertheless, the outcome shows that THF offers the best recovery, and it was used as the optimal elution solvent in subsequent tests.

**Figure 7:**
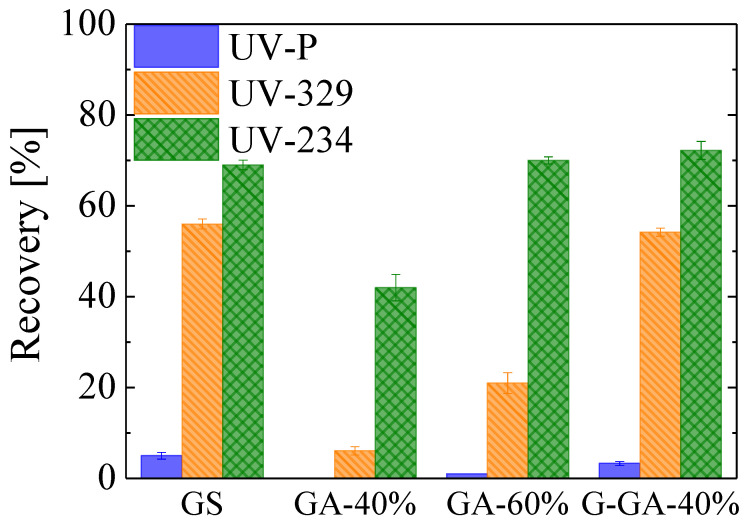
Extraction performance of GS and GAs using THF as the elution solvent in a fast configuration (approx. 1 min elution time, n = 3).

The SPE performance of the different synthesized aerogels was evaluated using THF after one minute of elution. The recoveries are summarized in Figure 7, and through them, the influence of both the structural (i.e., presence of graphenic domains) and the textural properties of the different materials was evaluated. GA-40% shows the poorest extraction capacity, with recoveries below 50%. G-GA-40% achieves the best performance among the aerogels, with similar recovery values as GS, while GA-60% presents an intermediate performance. These results show the great influence of the graphenic domains on the extraction performance. Compared to GS, it is clear that a decrease in the rGO content reduces the recovery achieved by GA-40% and GA-60%. However, competitive results were obtained by promoting the formation of more crystalline structures through the heat treatment at a higher temperature in G-GA-40% (Figure 2 and Figure 7). It is expected that this better crystallinity will improve the abovementioned interactions between the UV-benzotriazoles and the surface of the extractant, when compared with that of GA-40%. Indeed, the higher crystallinity detected by both XRD and Raman is a consequence of the improved perfection of the graphenic/graphitic structures of GA-40% that will enhance both dispersive and π-π interactions with target molecules. It should be mentioned that not only will the rearrangement of the rGO structures in the GA-40% aerogel contribute to the higher crystallinity of the G-GA-40%, but the extension of the graphitization to the surrounding RF-derived carbon, as promoted by these rGO structures, will as well [38].

On the other hand, the variation in the rGO content between GA-60% and GA-40% results in similar XRD profiles but different V_micro_ (i.e., 0.06 and 0.09 mL g^−1^, respectively, Table 1). Then, and in spite of the small micropore volumes of these samples, the higher performance of GA-60% could also be a consequence of the V_micro_ reduction hence the elimination of irreversible sorption sites. Therefore, high recovery values are reached by means of a high presence of graphenic domains and low V_micro_, characteristics that would contribute to the outstanding recovery of G-GA-40% (see Figure 7). When pondering both contributions, it is worth noting that the recoveries of GS are higher than those of GA-60%, even if their correspondent V_micro_ values are 0.08 and 0.06 mL g^−1^, respectively (Figure 7 and Table 1), which suggests that the rGO content of the material would be of greater relevance for the extraction performance. Finally, it was also observed that the macroporous character favors the permeability for all the materials, but only the morphological properties of GAs presented advantages, avoiding the compression of the material bed during the load and elution processes (Figure S2). From the results obtained in this section, GA-40% was discarded as a promising material for the extraction process, and the optimization of the proposed method was only carried out with GA-60% and G-GA-40%.

#### 3.2.2. Optimization of the SPE Method: Elution Time and Elution Volume

**Figure 8:**
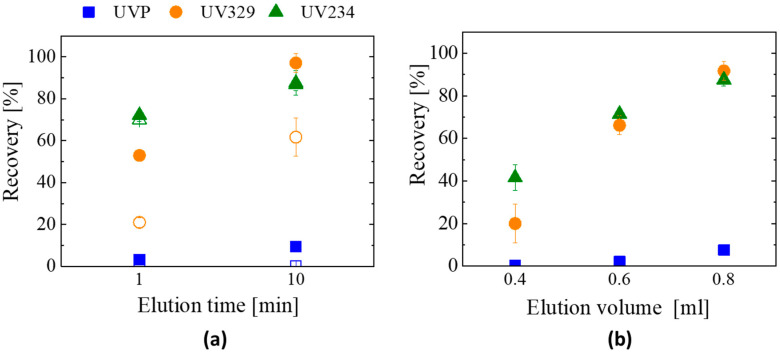
Effect of (**a**) elution time (empty symbols correspond to sample GA-60% and filled symbols correspond to G-GA-40%) and (**b**) recovery of UV-benzotriazoles in G-GA-40% cartridges using different elution volumes (n = 3).

In the extraction tests of the previous section, the cartridges were used as a syringe filter performing a rapid loading and elution SPE process. While keeping the same syringe filter configuration, the elution time was now increased up to approx. 10 min by decanting the solvent through the material bed by gravity (drop by drop), which increases the contact time and favors the interaction between the loaded material and the elution solvent [23,34,36]. Higher recovery percentages were then obtained for both GA-60% and G-GA-40% (Figure 8a): (i) when increasing the elution time, the recovery percentages of the different UV-benzotriazoles show the same trend for the two extractants, with significant differences only found for UV329; (ii) the recovery of UV234 increases from about 70 to 85% for both materials; (iii) UV329 is the most influenced compound, doubling (at least) its recovery percentage to 64% and 97% for GA-60% and G-GA-40%, respectively; and (iv) a slight increase was detected for UVP, but still below 10% of recovery. This behavior confirms that UV-benzotriazoles with a more polar character (e.g., UVP) are not retained in the material under the conditions tested, and only the less polar ones (e.g., UV234) can be successfully extracted using 10 min elution. Based on these results, G-GA-40% is the material with the highest potential for SPE, as it achieves the highest recovery rates (>80%) and better reproducibility compared to GA-60%.

To reach such performance with both aerogels, 0.8 mL of the solvent (and 10 min of elution time) was required. When compared to the reported SPE methodologies for the extraction of UV-benzotriazoles, which include very long extraction times and even the use of ultrasounds [23,34,36] (i.e., 60, 35 and 65 min, respectively), the rapid loading and elution of the rGO aerogel cartridges greatly benefit their implementation. Besides the limitation of these materials when working with small and more polar molecules (e.g., UVP), GAs are a highly competitive option when non-polar or large molecules are involved.

**Figure 9:**
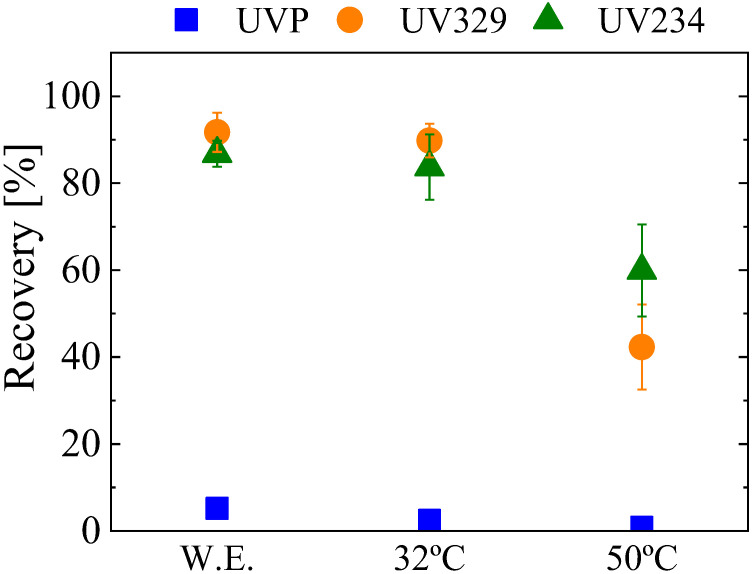
Effect of evaporation temperature on the SPE of the UV-benzotriazoles, using G-GA-40% as the extractant (n = 3). W.E. means that no evaporation step was carried out.

In addition to the recovery rate values, another very relevant parameter in SPE is the enrichment factor (EF), which quantifies the analyte concentration reached by the method. A theoretical (EF_theo_) and a real (EF_real_) enrichment factor can be defined. EF_theo_ can be improved by decreasing the volumes of the elution solvent (THF, in this case), as the ratio of the sample volume divided by the volume of the elution solvent would increase. However, the smaller the solvent volume, the lower the recovery [19,20], which makes necessary the optimization of this variable to achieve a competitive EF_real_, defined as EF_theo_ multiplied by the recovery. Figure 8b shows the recovery rates obtained with different elution volumes using G-GA-40% as the extractant in the SPE process. Recoveries of about 90% are reached using 0.8 mL of THF, which corresponds to an EF_real_ of about 11.2. In the opposite case, even if the EF_theo_ increases up to 25 when using 0.4 mL of the solvent, the poor recovery values (below 50% for all UV-benzotriazoles tested) make the EF_real_ only ~8. A similar behavior occurs with the intermediate point (i.e., 0.6 mL of elution solvent), where the resultant EF_real_ is 11.6. This means that the volumes tested have similar or lower EF_real_ than the one reached when using 0.8 mL of THF, thus making this strategy unfeasible for the enrichment of samples. As an alternative approach, the evaporation of the solvent and its subsequent restitution using smaller amounts (of solvent) was carried out. The extraction was performed as in previous tests (0.8 mL of elution solvent; 10 min elution time); then, the obtained solution was evaporated using two different temperatures (i.e., 32 or 50 °C); finally, 0.2 mL of fresh THF was added to the dried sample and stirred for 1.5 min to redissolve the extracted benzotriazoles. As shown in Figure 9, the quantity of UV-benzotriazoles remains the same as that without the evaporation step at 32 °C, which confirms the good preservation of the species at such temperature. On the other hand, using 50 °C for such evaporation step lowers the detected quantity of UV-benzotriazoles, thus indicating the degradation of these species at a higher temperature. It is worth noting that the EF_real_ was considerably improved to about 40–45 by using the 32 °C evaporation step.

### 3.3. Applicability of the SPE Method

The methodology developed was finally applied to extract UV-benzotriazoles mixtures from water at different concentrations, using SPE with GS, GA-60% and G-GA-40% as extractants. Mixtures of the three UV-benzotriazoles at 1, 5, 50 or 100 ng mL^−1^ in water were prepared and passed through the cartridges (10 mL), and the elution was performed with 0.8 mL of THF and 10 min of elution time in all cases. With the chromatographic methodology proposed (Section 2.3), the lower quantifiable concentration of UV329 and UV234 was approx. 2 ng mL^−1^ (Table 1). Then, to ensure a correct identification, just the eluted solutions from cartridges loaded with 5, 50 or 100 ng mL^−1^ were directly analyzed (EF_real_~11.4). In addition, the eluted samples when using lower loaded solutions (i.e., 1 ng mL^−1^) were evaporated and redissolved (32 °C and 0.2 mL of THF, respectively) to achieve the maximum enrichment factor (EF_real_~42.5). The resultant recoveries are summarized in Table 2. With respect to the different stationary phases, the behavior observed in the first extraction test (see Figure 7) remains the same, i.e., G-GA-40% presents a similar performance as the GS reference material. For example, the recovery values when testing solutions of 100 ng mL^−1^ show at least 80% of recovery for both UV329 and UV234. In the case of GA-60%, the lower quantity of graphenic structures makes the recovery of UV329 relatively low (61.7%), while UV324 is successfully recovered (87.6%). When solutions of concentrations <100 ng mL^−1^ are used for SPE, the expected trend implies a reduction in the recovery rate (Table 2). It is nonetheless worth mentioning that the GAs developed in this work bring about recoveries above 60% and 70% for GA-60% and G-GA-40%, respectively, at very low UV-benzotriazole concentrations (Table 2).

The reuse of selected GA-60% and G-GA-40% cartridges was tested by extracting, three consecutive times, a solution of the three UV-benzotriazoles in water (100 ng mL^−1^), washing the cartridges after each use (see Experimental Section 2.3 for additional details). The recovery values were almost identical in the three extractions (Figure S1), and the mechanical stability of the cartridges was outstanding. As for the carryover effect, it was also determined in both GA-60% and G-GA-40% cartridges after extracting 100 ng mL^−1^ solutions (Table 3). In the case of G-GA-40%, this effect was only noticeable, i.e., above the limit of detection of the HPLC method (25 ng mL^−1^, Table S1), for UV-234 (carryover of 5.2%; very close to the SD of the method; Table 2).

Considering the results of the reuse and carryover tests, it seems clear that the GAs require a heat treatment (as performed in the reuse tests) to remove the molecules that remain adsorbed on the extractant. This will be especially critical for the low-molecular-weight UV-benzotriazole UVP. For this particular benzotriazole (UVP), different organic solvents were used to elute the adsorbed quantities, with very limited success. Only dichloromethane (DCM) was able to remove a small amount (23.8%) of the UVP previously adsorbed. Nonetheless, it also seems clear that the adsorption sites of UVP have little interference in the adsorption of the two bigger molecules UV329 and UV234—UVP is mostly adsorbed on the micropores.

In order to directly quantify the original concentrations in the aqueous samples, the calibration of the SPE-HPLC method was finally carried out by extracting standard solutions of benzotriazoles (3–250 ng mL^−1^) through GA-60% and G-GA-40% prior to the HPLC-UV analysis. The results are collected in Table 4. Only the direct extraction method (i.e., without sample evaporation) was evaluated. The difference in the extraction performance of GA-60% with respect to G-GA-40% is observed in the fitting equations. The HPLC-UV peak intensities recorded in solutions extracted with GA-60% were smaller than the intensities recorded with standard solutions, which resulted in equations with lower slope values. Additionally, the LOD and LOQ values showed a slightly lower reproducibility (compared to G-GA-40%) when using this particular aerogel for the SPE-HPLC method.

To conclude this section, Table 5 shows some parameters regarding the performance of the rGO aerogels used for the extraction of UV-benzotriazoles in comparison with other graphene-based materials reported in the literature. The recoveries obtained in this work are very similar to those of the reference materials. However, differences in EF_theo_ values are remarkable, with the GAs comparing well even with the methodology proposed by Zhang et al. [23], which is considered an advanced extraction technology (i.e., stir bar sorptive extraction or SBSE). In addition, rGO aerogels show additional advantages when compared to the advanced GSs, in terms of SPE elution times (only 10 min in our case) and materials preparation. In terms of the latter aspect, it should be noted that the GAs were obtained following a quick route assisted by microwave heating that lasted only 3 days, and it provided materials with enhanced mechanical integrity and easier handling (Figure S2).

### 3.4. SPE-HPLC Tests with Real (River) Waters

The sample obtained from the Nora river and the spiked samples were extracted with both GA-60% and G-GA-40%. The results obtained are collected in Table 6. Benzotriazoles were not detected in the river water. The benzotriazole concentrations found in the eluted samples (and, hence, the recoveries) were calculated considering the concentration of standard solutions of benzotriazoles in THF (Table S1). The matrix effect (ME, %), for a given benzotriazole, was also determined as follows:

ME (%) = [(I_spiked sample_ − I_river sample_)/I_standard SPE-HPLC_] × 100 (1)

with I_spiked sample_ and I_river sample_ corresponding to the HPLC-UV intensity of the SPE-spiked and non-spiked river samples, respectively, and I_standard SPE-HPLC_ corresponding to the HPLC-UV intensity of a benzotriazole solution in MilliQ^®^ water with a concentration identical to the spike, after extraction with the SPE method (Table 4). In this way, we could evaluate the possible amplification of or reduction in the HPLC-UV signal of a given benzotriazole due to the presence of different molecules in the river water. The closer the ME values were to 100%, the lower the matrix effect. According to the results of Table 6, the recoveries showed differences in the extraction performance of both aerogels, whereas the ME values pointed out that the extractants maintain their performance reasonably well when working with real (river) samples.

## 4. Conclusions

The SPE method developed in this work showed a good recovery and reproducibility for the extraction of UV-benzotriazoles in water samples in a range of concentration between 1 and 100 ng mL^−1^. In the extraction of UV-benzotriazoles, several of the GAs showed an excellent performance that achieved recoveries over 80%, similar to the recovery values reported for GSs. The inclusion of carbonized RF polymeric species in the GAs renders a tougher structure which minimizes the compression of the extraction bed during the loading and elution processes. Since the presence of the RF polymeric networks in the GAs reduces the graphenic domains available for interaction with the UV-benzotriazoles, a key aspect of this work was to find the optimal quantity of rGO required in the GAs to obtain extractants with acceptable performances. Hence, rGO contents of about 40 wt.% and 60 wt.% were enough for the development of functional materials for SPE. In terms of the SPE method, all the graphene-based materials showed fast kinetics for the adsorption and desorption of larger molecules with a non-polar character (i.e., UV329 and UV234), taking only 10 min to perform extractions with high recoveries. Based on the ideal volume of the elution solvent required for a good recovery (i.e., 0.8 mL), the theoretical enrichment factor of the method is 12.5, which was improved up to 50 by the evaporation (32 °C) of the elution solvent. This capacity for the sample concentration, in combination with the chromatographic methodology proposed, rendered a detection limit of 1 ng mL^−1^. The two selected aerogels were also tested with river waters, keeping their extraction performance and showing a low matrix effect. The use of the GAs is thus a cost-effective option that brings simplicity and speed to the extraction process using graphene-based materials. GAs represent an attractive alternative compared to GSs due to the simple and faster procedure and the lower amount of GO used in their synthesis.

## Figures and Tables

**Table 1:** Textural properties of the graphene-based materials.

Material	S_BET_ (m^2^ g^−1^)	V_total_ (mL g^−1^)	V_micro_^a^ (mL g^−1^)
GS	203	0.89	0.08
GA-40%	247	0.41	0.09
GA-60%	164	0.49	0.06
G-GA-40%	51	0.26	0.01

^a^ Dubinin–Radushkevich model.

**Table 2:** Recoveries of the proposed method when testing samples of different concentrations in water.

Material	Concentration (ng mL^−1^)	EF_theo_	UV329		UV234	
			Recovery (%)	SD ^a^ (%)	Recovery (%)	SD ^a^ (%)
GS	1	50	83.6	10.4	76.7	5.0
	100	12.5	91.0	7.8	79.7	6.8
GA-60%	1	50	70.4	8.7	84.9	2.7
	5	12.5	56.6	5.4	88.1	4.4
	100	12.5	61.7	9.1	87.6	5.8
G-GA-40%	1	50	78.2	9.9	82.6	7.2
	5	12.5	93.2	5.8	96.1	9.2
	50	12.5	80.1	8.1	83.9	3.2
	100	12.5	97.1	4.5	88.6	4.2

^a^ Standard deviation.

**Table 3:** Carryover effect on the SPE extraction of UV-benzotriazoles aqueous mixtures (100 ng mL^−1^), using 0.8 mL THF as the elution solvent.

Material	UVP		UV329		UV234	
	Recovery (%)	SD ^a^ (%)	Recovery (%)	SD ^a^ (%)	Recovery (%)	SD ^a^ (%)
GA-60%	N.D. ^b^	-	9.7	3.2	5.2	3.9
G-GA-40%	N.D. ^b^	-	N.D. ^b^	-	4.7	2.1

^a^ Standard deviation. ^b^ Not detected.

**Table 4:** Analytical features of the proposed SPE-HPLC method for the quantitative analysis of UV-benzotriazoles using GA-60% and G-GA-40%.

Sample	Analyte	Range (ng mL^−1^)	Equation	R^2^	LOD ^a^ (ng mL^−1^)	LOQ ^b^ (ng mL^−1^)
GA-60%	UV329	3–250	y = 0.215x + 2.329	0.9960	1.43	4.76
	UV234	3–250	y = 0.317x + 0.072	0.9948	1.16	3.88
G-GA-40%	UV329	3–250	y = 0.391x + 0.118	0.9981	0.95	3.17
	UV234	3–250	y = 0.340x + 0.338	0.9998	0.95	3.18

^a^ Limit of detection. ^b^ Limit of quantification.

**Table 5:** Performance of the GAs compared to that of other graphene-based materials.

Method	Extractant		Performance Parameters			Ref.
	Material	Time of Synthesis (Days)	Extraction Time (min)	Recovery (%)	EF_theo_	
SPE-HPLC-UV	GS	4.1	15	89–105	6.25	[21]
SBSE-HPLC-DAD	GS/Ni coated	>7	60	84–112	67	[23]
SPE-HPLC-UV	GAs	3	10	75–97	12.5–50	This work

**Table 6:** Results for Nora river water using the proposed SPE-HPLC method with GA-60% and G-GA-40% cartridges (n = 3).

Material	Spiked (ng mL^−1^)	UV329			UV234		
		Found (ng mL^−1^)	Recovery (SD ^a^) (%)	ME ^b^ (SD ^a^) (%)	Found (ng mL^−1^)	Recovery (SD ^a^) (%)	ME ^b^ (SD ^a^) (%)
GA-60%	0	N.D. ^c^	-	-	N.D. ^c^	-	-
	25	20.05	80.0 (9.5)	98.8 (7.1)	21.89	87.6 (3.02)	89.8 (1.95)
	50	34.74	69.5 (8.4)	97.8 (6.4)	35.95	72.9 (2.26)	80.8 (1.21)
	100	68.55	68.6 (5.9)	88.0 (5.2)	84.61	84.6 (8.2)	91.9 (8.8)
G-GA-40%	0	N.D. ^c^	-	-	N.D. ^c^	-	-
	25	20.88	83.5 (1.5)	81.4 (1.5)	21.47	85.9 (2.8)	93.7 (2.6)
	50	46.93	93.9 (4.9)	87.4 (4.1)	51.36	102.7 (3.3)	97.6 (3.2)
	100	88.31	88.3 (4.9)	90.6 (4.9)	81.45	81. 5 (9.7)	83.5 (7.5)

^a^ Standard deviation. ^b^ Matrix effect. ^c^ Not detected.

## Data Availability

The data presented in this study are available on request from the corresponding author. The data are not publicly available due to privacy reasons.

## Supplementary Materials

The following supporting information can be downloaded at: https://www.mdpi.com/article/10.3390/ma16062519/s1, Figure S1: Recovery values of (a) GA-60% and (b) G-GA-40% cartridges after three consecutive reuses (see Experimental Section 2.3 for details); Figure S2: Physical aspect of GS and GA-60% SPE cartridges after the loading and elution of samples. The GS sample is irreversibly compacted after use, whereas the GA-60% preserves the mechanical integrity; Table S1: Analytical features of the proposed HPLC method for the quantitative analysis of UV-benzotriazoles dissolved in the best extraction solvent (THF); Table S2: Properties of the UV-benzotriazoles used in this study.

## References

[1] Dwivedi A. (2017). Researches in Water Pollution: A Review. Int. Res. J. Nat. Appl. Sci..

[2] Chaudhry F., Malik M. (2017). Factors Affecting Water Pollution: A Review. J. Ecosyst. Ecography.

[3] Yang Y., Zhang X., Jiang J., Han J., Li W., Li X., Yee Leung K.M., Snyder S.A., Alvarez P.J.J. (2022). Which Micropollutants in Water Environments Deserve More Attention Globally?. Environ. Sci. Technol..

[4] Speltini A., Pastore M., Merlo F., Maraschi F., Sturini M., Dondi D., Profumo A. (2019). Humic Acids Pyrolyzed onto Silica Microparticles for Solid-Phase Extraction of Benzotriazoles and Benzothiazoles from Environmental Waters. Chromatographia.

[5] Kim M.-K., Zoh K.-D. (2016). Occurrence and Removals of Micropollutants in Water Environment. Environ. Eng. Res..

[6] Rogowska J., Cieszynska-Semenowicz M., Ratajczyk W., Wolska L. (2020). Micropollutants in Treated Wastewater. Ambio.

[7] Egambaram O.P., Kesavan Pillai S., Ray S.S. (2020). Materials Science Challenges in Skin UV Protection: A Review. Photochem. Photobiol..

[8] Kiejza D., Karpińska J., Kotowska U. (2022). Degradation of Benzotriazole UV Stabilizers in PAA/d-Electron Metal Ions Systems—Removal Kinetics, Products and Mechanism Evaluation. Molecules.

[9] Alotaibi M.D., McKinley A.J., Patterson B.M., Reeder A.Y. (2015). Benzotriazoles in the Aquatic Environment: A Review of Their Occurrence, Toxicity, Degradation and Analysis. Water Air Soil Pollut..

[10] Kraševec I., Prosen H. (2018). Solid-Phase Extraction of Polar Benzotriazoles as Environmental Pollutants: A Review. Molecules.

[11] Nouri N., Khorram P., Duman O., Sibel T., Hassan S. (2020). Overview of Nanosorbents Used in Solid Phase Extraction Techniques for the Monitoring of Emerging Organic Contaminants in Water and Wastewater Samples. Trends Environ. Anal. Chem..

[12] Poole C.F. (2020). Core Concepts and Milestones in the Development of Solid-Phase Extraction. Solid-Phase Extraction.

[13] Leusch F., Prochazka E., Carswell S., Escher B. Optimising Micropollutants Extraction for Analysis of Water Samples: Comparison of Different Solid Phase Materials and Liquid-Liquid Extraction. Proceedings of the Science Forum and Stakeholder Engagement: Building Linkages, Collaboration and Science Quality.

[14] Guillot S., Kelly M.T., Fenet H., Larroque M. (2006). Evaluation of Solid-Phase Microextraction as an Alternative to the Official Method for the Analysis of Organic Micro-Pollutants in Drinking Water. J. Chromatogr. A.

[15] Fernández-Fernández V., Ramil M., Cela R., Rodríguez I. (2022). Solid-Phase Extraction and Fractionation of Multiclass Pollutants from Wastewater Followed by Liquid Chromatography Tandem-Mass Spectrometry Analysis. Anal. Bioanal. Chem..

[16] Čizmić M., Babić S., Kaštelan-Macan M. (2017). Multi-Class Determination of Pharmaceuticals in Wastewaters by Solid-Phase Extraction and Liquid Chromatography Tandem Mass Spectrometry with Matrix Effect Study. Environ. Sci. Pollut. Res..

[17] Masini J.C., do Nascimento F.H., Vitek R. (2021). Porous Monolithic Materials for Extraction and Preconcentration of Pollutants from Environmental Waters. Trends Environ. Anal. Chem..

[18] Zheng X., Zhao Y., Wen W., Zheng H., Gao L. (2021). Application of Graphene and Its Compounds in Pretreatment of Environmental Samples. IOP Conf. Ser. Earth Environ. Sci..

[19] Shi Z., Li Q., Xu D., Huai Q., Zhang H. (2016). Graphene-Based Pipette Tip Solid-Phase Extraction with Ultra-High Performance Liquid Chromatography and Tandem Mass Spectrometry for the Analysis of Carbamate Pesticide Residues in Fruit Juice: Sample Preparation. J. Sep. Sci..

[20] Han Q., Liang Q., Zhang X., Yang L., Ding M. (2016). Graphene Aerogel Based Monolith for Effective Solid-Phase Extraction of Trace Environmental Pollutants from Water Samples. J. Chromatogr. A.

[21] Wang X., Wang J., Du T., Kou H., Du X., Lu X. (2018). Determination of Six Benzotriazole Ultraviolet Filters in Water and Cosmetic Samples by Graphene Sponge-Based Solid-Phase Extraction Followed by High-Performance Liquid Chromatography. Anal. Bioanal. Chem..

[22] Pena-Pereira F., Romero V., de la Calle I., Lavilla I., Bendicho C. (2021). Graphene-Based Nanocomposites in Analytical Extraction Processes. TrAC Trends Anal. Chem..

[23] Zhang Q., You L., Chen B., He M., Hu B. (2021). Reduced Graphene Oxide Coated Nickel Foam for Stir Bar Sorptive Extraction of Benzotriazole Ultraviolet Absorbents from Environmental Water. Talanta.

[24] Li N., Jiang H.-L., Wang X., Wang X., Xu G., Zhang B., Wang L., Zhao R.-S., Lin J.-M. (2018). Recent Advances in Graphene-Based Magnetic Composites for Magnetic Solid-Phase Extraction. TrAC Trends Anal. Chem..

[25] dos Santos-Gómez L., García J.R., Montes-Morán M.A., Menéndez J.A., García-Granda S., Arenillas A. (2021). Ultralight-Weight Graphene Aerogels with Extremely High Electrical Conductivity. Small.

[26] Park S., An J., Potts J.R., Velamakanni A., Murali S., Ruoff R.S. (2011). Hydrazine-Reduction of Graphite- and Graphene Oxide. Carbon.

[27] Huh S.H. (2011). Thermal Reduction of Graphene Oxide. Physics and Applications of Graphene-Experiments.

[28] Chatzimitakos T.G., Stalikas C.D. (2020). Sponges and Sponge-Like Materials in Sample Preparation: A Journey from Past to Present and into the Future. Molecules.

[29] Thommes M., Kaneko K., Neimark A.V., Olivier J.P., Rodriguez-Reinoso F., Rouquerol J., Sing K.S.W. (2015). Physisorption of Gases, with Special Reference to the Evaluation of Surface Area and Pore Size Distribution (IUPAC Technical Report). Pure Appl. Chem..

[30] Moreno A.H., Arenillas A., Calvo E.G., Bermúdez J.M., Menéndez J.A. (2013). Carbonisation of Resorcinol–Formaldehyde Organic Xerogels: Effect of Temperature, Particle Size and Heating Rate on the Porosity of Carbon Xerogels. J. Anal. Appl. Pyrolysis.

[31] Rey-Raap N., Angel Menéndez J., Arenillas A. (2014). Simultaneous Adjustment of the Main Chemical Variables to Fine-Tune the Porosity of Carbon Xerogels. Carbon.

[32] Canal-Rodríguez M., Ramírez-Montoya L.A., Villanueva S.F., Flores-López S.L., Angel Menéndez J., Arenillas A., Montes-Morán M.A. (2019). Multiphase Graphitisation of Carbon Xerogels and Its Dependence on Their Pore Size. Carbon.

[33] Liu R., Ruan T., Wang T., Song S., Guo F., Jiang G. (2014). Determination of Nine Benzotriazole UV Stabilizers in Environmental Water Samples by Automated On-Line Solid Phase Extraction Coupled with High-Performance Liquid Chromatography–Tandem Mass Spectrometry. Talanta.

[34] Yu H., Di S., Su X., Wang J., Ning T., Yang H., Zhu S. (2022). Preparation of Beta-Cyclodextrin Based Nanocomposite for Magnetic Solid-Phase Extraction of Organic Ultraviolet Filters. J. Chromatogr. A.

[35] Yang F., Li X., Meng D., Yang Y. (2017). Determination of Ultraviolet Absorbers and Light Stabilizers in Food Packaging Bags by Magnetic Solid Phase Extraction Followed by High-Performance Liquid Chromatography. Food Anal. Methods.

[36] Montesdeoca-Esponda S., Sosa-Ferrera Z., Kabir A., Furton K.G., Santana-Rodríguez J.J. (2015). Fabric Phase Sorptive Extraction Followed by UHPLC-MS/MS for the Analysis of Benzotriazole UV Stabilizers in Sewage Samples. Anal. Bioanal. Chem..

[37] Zilfidou E., Kabir A., Furton K., Samanidou V. (2018). Fabric Phase Sorptive Extraction: Current State of the Art and Future Perspectives. Separations.

[38] Canal-Rodríguez M., Arenillas A., Villanueva S.F., Montes-Morán M.A., Menénedez J.A. (2020). Graphitized carbon xerogels for lithium-ion batteries. Materials.

