# Supplementary material for: Reduced Graphene Oxide Aerogels Cartridges for Solid Phase Extraction of Benzotriazoles
Source: Materials (Basel). 2023 Mar 22;16(6):2519. doi: 10.3390/ma16062519 (PMC10052701; doi:10.3390/ma16062519)
Supplement: Supplementary file 1 [file materials-16-02519-s001.zip › materials-2210653-supplementary.pdf]

Reduced Graphene Oxide Aerogels Cartridges for Solid Phase Extraction of Benzotriazoles

Samantha L. Flores-López [1], Ana Arenillas [1], Ivan Mikšík [2], J. Angel Menéndez [1] and Miguel A. Montes-Morán [1,*]

[1] Instituto de Ciencia y Tecnología del Carbono, INCAR-CSIC, Francisco Pintado Fe, 26, 33011 Oviedo, Spain

[2] Institute of Physiology, The Czech Academy of Sciences, 142 20 Prague, Czech Republic

Supplementary Material

Table S1. Analytical features of the proposed HPLC method for the quantitative analysis of UV-benzotriazoles dissolved in the best extraction solvent (THF).

Analyte	Range (ng mL^{-1})	Equation	R^2	LOQ [b] (ng mL^{-1})	LOD [a] (ng mL^{-1})
UVP	25-4000	$y = 1.45\ 10^{-6}x^2+0.0424x-1.2644$	0.9990	2.23	1.1
UV329	25-4000	$y = 1.16\ 10^{-6}x^2+0.0256x-0.0503$	0.9998	2.44	0.95
UV234	25-4000	$y = 7.63\ 10^{-7}x^2+0.0201x+0.0405$	0.9992	2.13	0.82

[a] Limit of detection. [b] Limit of quantification

Table S2. Properties of the UV-benzotriazoles used in this study.

Benzotriazole	Molecule	Molecular Weight	Log K_{ow}
UVP	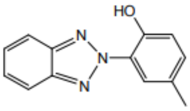	225.2	4.33
UV329	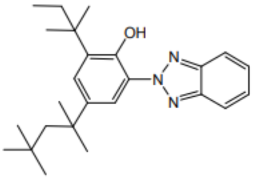	323.4	6.21
UV234	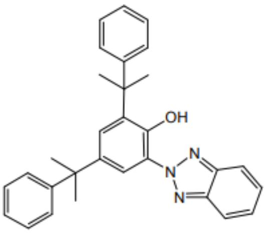	447.57	7.67

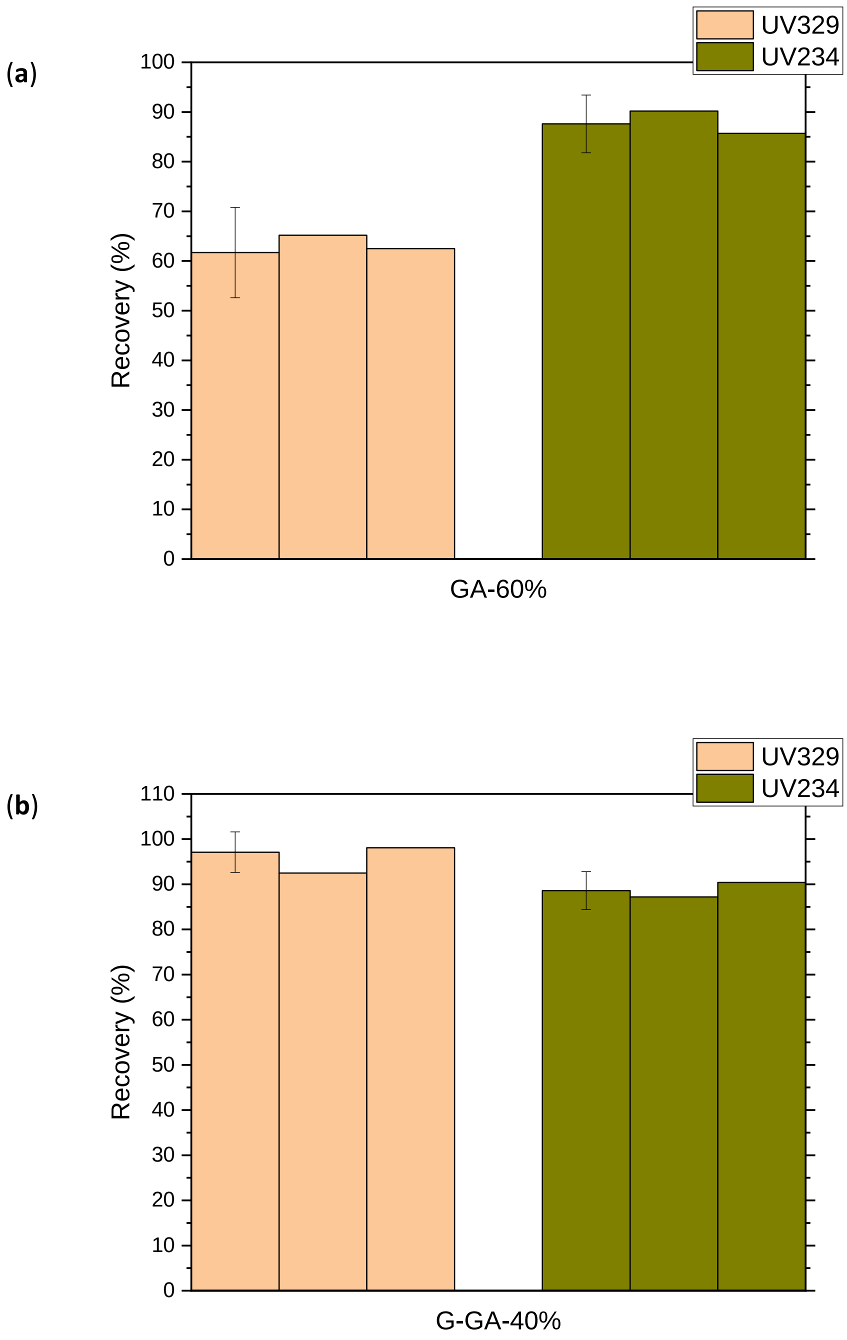

Figure S1. Recovery values of (**a**) GA-60% and (**b**) G-GA-40% cartridges after three consecutive reuses (see Experimental Section 2.3 for details).

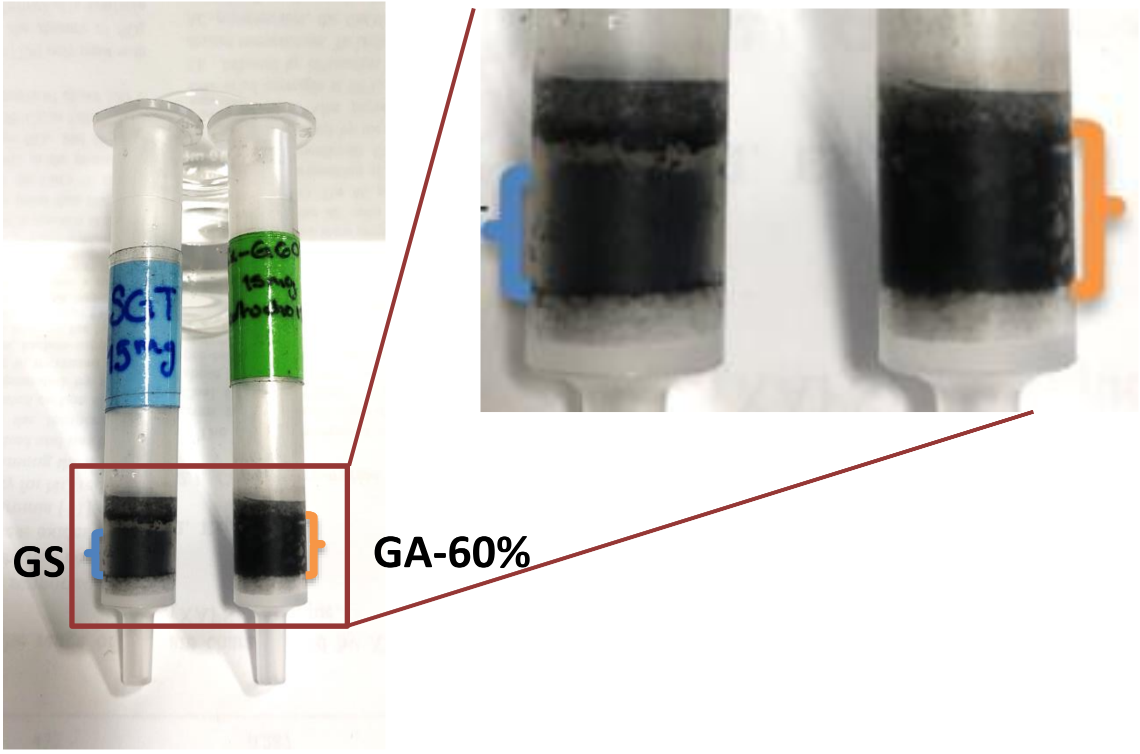

Figure S2. Physical aspect of GS and GA-60% SPE cartridges after loading and elution of samples. The GS sample is irreversibly compacted after use whereas the GA-60% preserves the mechanical integrity.
